# Isocitrate binds to the itaconic acid–responsive LysR-type transcriptional regulator RipR in *Salmonella* pathogenesis

**DOI:** 10.1016/j.jbc.2022.102562

**Published:** 2022-10-02

**Authors:** Nayeon Ki, Jinshil Kim, Inseong Jo, Yongseong Hyun, Sangryeol Ryu, Nam-Chul Ha

**Affiliations:** 1 Department of Food and Animal Biotechnology, Department of Agricultural Biotechnology, and Research Institute of Agriculture and Life Sciences, Seoul National University, Seoul, Republic of Korea; 2 Center for Food and Bioconvergence, Seoul National University, Seoul, Republic of Korea

**Keywords:** itaconic acid, LysR-type transcriptional regulator, isocitrate, glyoxylate cycle, 3-phenylpropionic acid, DBD, DNA-binding domain, ITC, isothermal titration calorimetry, LTTR, LysR-type transcriptional regulator, qRT-PCR, quantitative real-time PCR, RD, regulatory domain, TCA, tricarboxylic acid

## Abstract

Macrophages produce itaconic acid in phagosomes in response to bacterial cell wall component lipopolysaccharide to eliminate invading pathogenic bacteria. Itaconic acid competitively inhibits the first enzyme of the bacterial glyoxylate cycle. To overcome itaconic acid stress, bacteria employ the bacterial LysR-type transcriptional regulator RipR. However, it remains unknown which molecule activates RipR in bacterial pathogenesis. In this study, we determined the crystal structure of the regulatory domain of RipR from the intracellular pathogen *Salmonella*. The RipR regulatory domain structure exhibited the typical dimeric arrangement with the putative ligand-binding site between the two subdomains. Our isothermal titration calorimetry experiments identified isocitrate as the physiological ligand of RipR, whose intracellular level is increased in response to itaconic acid stress. We further found that 3-phenylpropionic acid significantly decreased the resistance of the bacteria to an itaconic acid challenge. Consistently, the complex structure revealed that the compound is antagonistically bound to the RipR ligand-binding site. This study provides the molecular basis of bacterial survival in itaconic acid stress from our immune systems. Further studies are required to reveal biochemical activity, which would elucidate how *Salmonella* survives in macrophage phagosomes by defending against itaconic acid inhibition of bacterial metabolism.

Macrophages are activated upon inflammatory signals, such as the bacterial cell wall component lipopolysaccharide, and kill the invading bacteria in their phagosome with diverse antimicrobial substances, such as H_2_O_2_ and HOCl (1, 2). Itaconic acid (2-methylenesuccinic acid), an unsaturated dicarboxylic acid, was recently noted as a new antibacterial substance in the macrophage phagosome (3). Itaconic acid is generated from the precursor *cis*-aconitate by the enzymatic activity of the immune-responsive gene 1 (Irg1) gene product, which is the most highly upregulated by the bacterial cell wall component lipopolysaccharide in macrophages (3, 4, 5, 6, 7, 8, 9). The antimicrobial activity of itaconic acid results from the inhibition of bacterial isocitrate lyase, which catalyzes the conversion of isocitrate to glyoxylate and succinate as the key enzyme in the glyoxylate shunt pathway or the glyoxylate cycle (10). The glyoxylate cycle is widely found in bacteria, protists, fungi, and plants as a variation of the tricarboxylic acid (TCA) cycle (11, 12). The glyoxylate cycle saves two carbons by bypassing the two decarboxylation reactions (isocitrate to α-ketoglutarate and α-ketoglutarate to succinyl-CoA) in the TCA cycle and thus is usually employed under limiting carbon source conditions (13).

Gram-negative bacteria have LysR-type transcriptional regulators (LTTRs) to properly regulate gene expression by recognizing signaling molecules in response to diverse environmental and physiological stimuli (14). LTTRs consist of the N-terminal DNA-binding domain (DBD) and the C-terminal regulatory domain (RD) exhibiting typical asymmetric tetrameric assembly (15). Binding of the cognate ligands to the RD triggers the changes in transcriptional activity *via* structural changes in the tetrameric assembly (16, 17). The LTTR RipR was identified as the vital component of the resistance to the antimicrobial function of itaconic acid in many gram-negative pathogenic bacteria (18). RipR activated the expression of the three enzymes *ripC*, *ripB*, and *ripA*, which degrade itaconic acid in many other pathogens including *Yersinia pestis*, *Pseudomonas aeruginosa*, *Mycobacterium tuberculosis*, and *Salmonella enterica* (3, 19, 20). In *S. enterica*, the drastic upregulation of *ripCBA* by itaconic acid was observed through a GFP reporter assay (18). RipR contributed to the bacterial resistance to itaconic acids in *S. enterica* and *Y. pestis* (18, 19). RipR has DBD and RD similar to those of other LTTRs and is expected to form a tetrameric assembly similar to that of like typical LTTRs.

This study focuses on the food poisoning bacterium *S. enterica*, which can survive in macrophage phagosomes through diverse mechanisms (21, 22, 23). *S. enterica* has the transcriptional activator RipR to cope with the itaconic acid stress present in macrophage phagosomes. However, it remains unknown which molecule activates RipR in the defense against the itaconic acid challenge in the macrophage phagosomes. Herein, we determine the crystal structure of the RipR RD and discover the cognate ligand molecules of RipR. We further found that plant-derived secondary metabolites inhibit the RipR function.

## Results

### Crystal structure of *S*. Typhimurium ligand-free RipR RD

**Figure 1:**
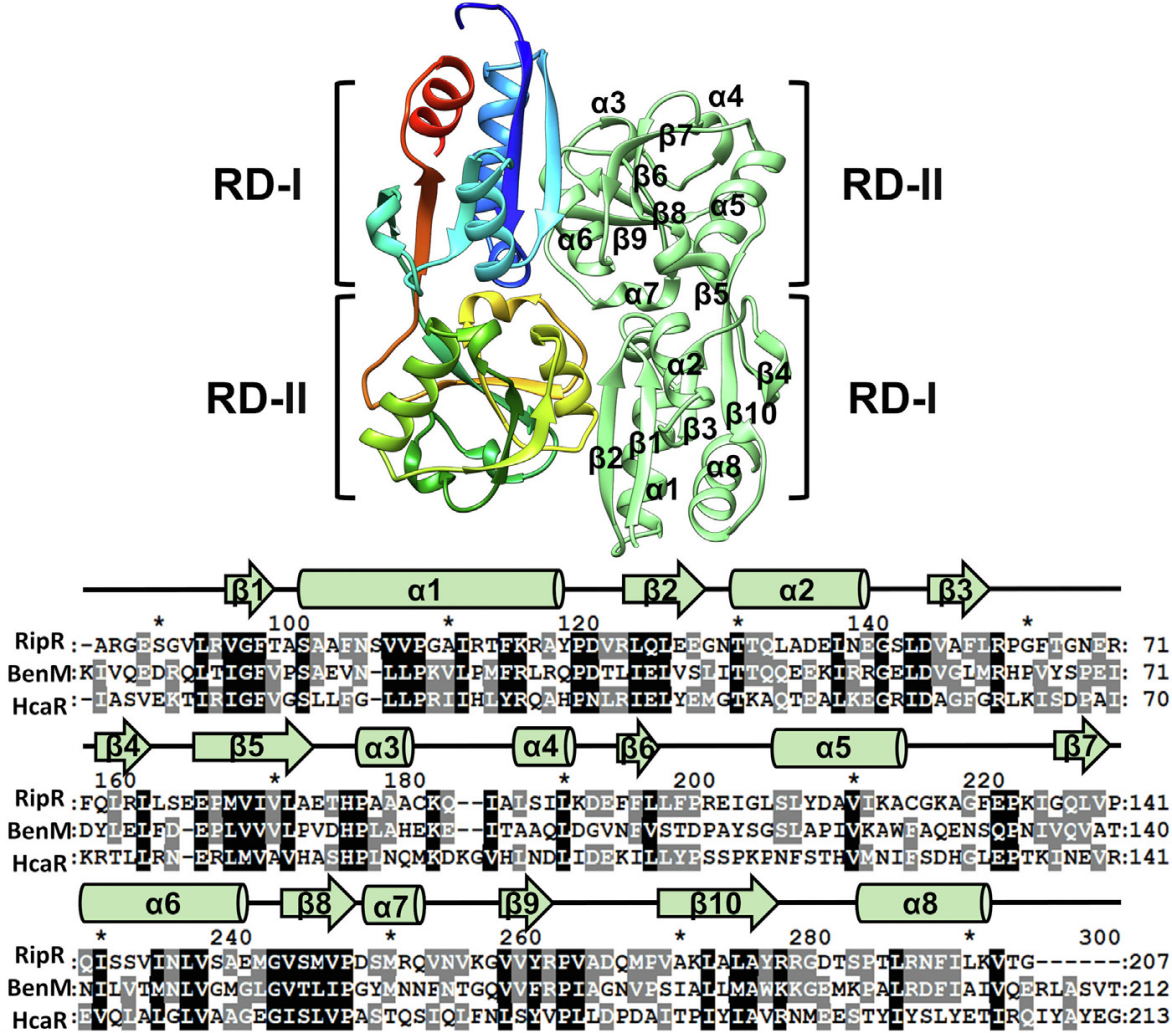
**Overall structure of the RipR RD dimer.** The asymmetric unit of the RipR RD from *S.* Typhimurium in the ribbon representations. The asymmetric unit contains two copies of the RipR RD. The protomer of RipR RD on the *left side* is depicted in *rainbow* color, while the other protomer on the *right side* is depicted in *pale green*. The subdomains RD-I and RD-II are labeled. RD-I comprises three α-helices (α1, α2, and α8), and five β-strands (β1, β2, β3, β4, and β10). RD-II comprises five α-helices (α3, α4, α5, α6, and α7) and five β-strands (β5, β6, β7, β8, and β9). Sequence comparison of RipR to its homologs: BenM, *Acinetobacter baylyi* and HcaR, *Escherichia coli*. The secondary structural elements are annotated above the sequence. RD, regulatory domain.

We expressed the RD (residues 87–292) of RipR from *S. enterica* serovar Typhimurium (*S*. Typhimurium) in the *Escherichia coli* expression system. The purified protein was crystallized in the space group of *P2*_*1*_, and its crystal structure was determined at 2.3 Å resolution by the molecular replacement method. The search model for the molecular replacement was generated by the structural prediction program Alphafold 2 (24, 25). The asymmetric unit contained four closely interacting protomers, indicating two homodimeric assemblies of the RipR RD, similar to typical LTTR RDs (16, 17, 26, 27, 28, 29) (Fig. 1). Size-exclusion chromatography with multiangle light scattering confirmed the homodimer in the solution state, as observed in many LTTR RDs (Fig. S1).

**Figure 2:**
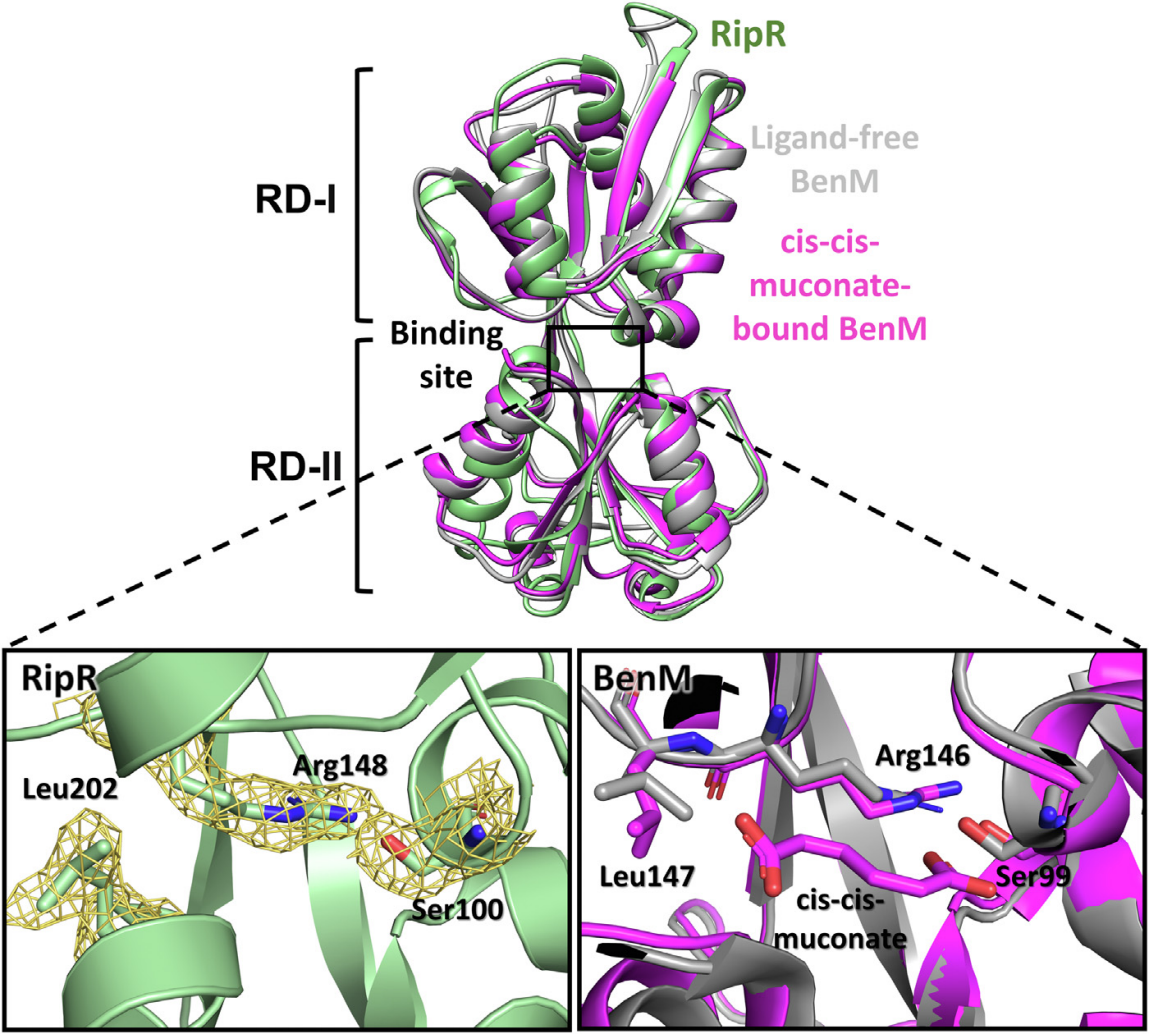
**Structural comparison of RipR RD to ligand-free and ligand-bound BenM RDs.** A protomer of the Rip RD (*pale green*) was superposed on BenM RD from *Acinetobacter baylyi* (PDB code, 2F6G) representing the ligand-free form (rmsd = 1.536 Å) and on *cis*-*cis*-muconate–bound BenM RD (PDB code, 2F7A; rmsd = 1.571 Å). The *red rectangle* indicates the putative ligand-binding site, which is enlarged with the conserved residues involved in the ligand binding. The *cis*-*cis*-muconate is also depicted in stick representations in the *bottom right*. The electron density map of RipR is shown in *yellow*. RD, regulatory domain.

Similar to typical LTTR RDs, the RipR RD is further divided into the two subdomains RD-I and RD-II. RD-I from one protomer interacts with RD-II from the other protomer in the homodimeric arrangement (Fig. 1*A*). The RD of the benzoate and *cis*-*cis*-muconate–responsive LTTR BenM was discovered as the top structural ortholog of RipR RD using the FoldSeek server (rmsd 1.536 Å; Table 1), which is a specialized program to search the structural homologs by sensitive comparisons of large structure sets (30). The structural superposition on the ligand-bound structures of the BenM RD suggested the putative ligand-binding site of RipR RD in the space between the two subdomains RD-I and RD-II (Fig. 2). Our RipR RD structure seemed to be a ligand-free structure, since no extra electron density map was found in the putative ligand-binding pocket of RipR RD.

**Table 1:** Results of a Foldseek search with the RipR structure

PDB ID	Score	E-values	Sequence identity (%)	Gene ID	Description	Oligomer state
2H9B	787	4.292e-20	25.4	BenM from *Acinetobacter baylyi* ADP1 (R156H/T157S)	LysR-type transcriptional regulator	Homodimer
2F78	775	9.231e-20	25.7	BenM with its effector benzoate from *Acinetobacter baylyi*	LysR-type transcriptional regulator	Homodimer
2F7A	772	1.118e-19	25.7	BenM with its effector *cis*, *cis*-muconate from *Acinetobacter baylyi*	LysR-type transcriptional regulator	Homodimer
2H99	769	1.354e-19	25.7	BenM from *Acinetobacter baylyi* ADP1 (R156H, T157S)	LysR-type transcriptional regulator	Homodimer
2F97	764	1.863e-19	25.7	BenM from *Acinetobacter baylyi* ADP1 (high pH)	LysR-type transcriptional regulator	Homodimer
2F6G	755	3.308e-19	24.7	BenM from *Acinetobacter baylyi* ADP1	LysR-type transcriptional regulator	Homodimer
3GLB	753	3.758e-19	24.7	CatM from *Acinetobacter baylyi* ADP1 (R156H)	LysR-type transcriptional regulator	Homodimer
2H98	742	7.584e-19	25.7	CatM from *Acinetobacter baylyi* ADP1 (V158M)	LysR-type transcriptional regulator	Homodimer
3K1N	741	8.084e-19	25.7	BenM from *Acinetobacter baylyi* ADP1 (full-length)	LysR-type transcriptional regulator	Homodimer
3K1P	733	1.347e-18	24.6	BenM from *Acinetobacter baylyi* ADP1 (E226K) (full-length)	LysR-type transcriptional regulator	Homodimer
2F7C	727	1.975e-18	24.7	CatM with its effector *cis*, *cis*-muconate from *Acinetobacter baylyi*	LysR-type transcriptional regulator	Homodimer
1IXC	689	2.233e-17	21.6	CbnR from *Cupriavidus necator*	LysR-type transcriptional regulator	Homotetramer
6G1D	688	2.380e-17	24.1	OxyR from *Corynebacterium glutamicum*	LysR-type transcriptional regulator	Homotetramer
2HXR	680	3.966e-17	23	CynR from *Escherichia coli K-12*	Probable transcriptional regulator	Homodimer
5TED	678	4.505e-17	21.9	QuiR with shikimate from *Listeria monocytogenes* EGD-e	LysR-type transcriptional regulator	Homotrimer

The putative ligand-binding sites of RipR RD were lined with Ser100, Arg148, and Leu202 (Fig. 2), which are mostly conserved among the RipR homologs and BenM (31). In particular, the Arg residue corresponding to Arg148 makes the ionic interaction with the carboxylic acid of the bound *cis*-*cis*-muconate in the BenM RD (PDB code: 2F7A; Fig. 2). Thus, these findings suggest that the cognate ligand of the RipR RD contains carboxylic acid moieties similar to those in the BenM RD ligand *cis*-*cis*-muconate.

### Isocitrate as the cognate ligand of RipR

**Figure 3:**
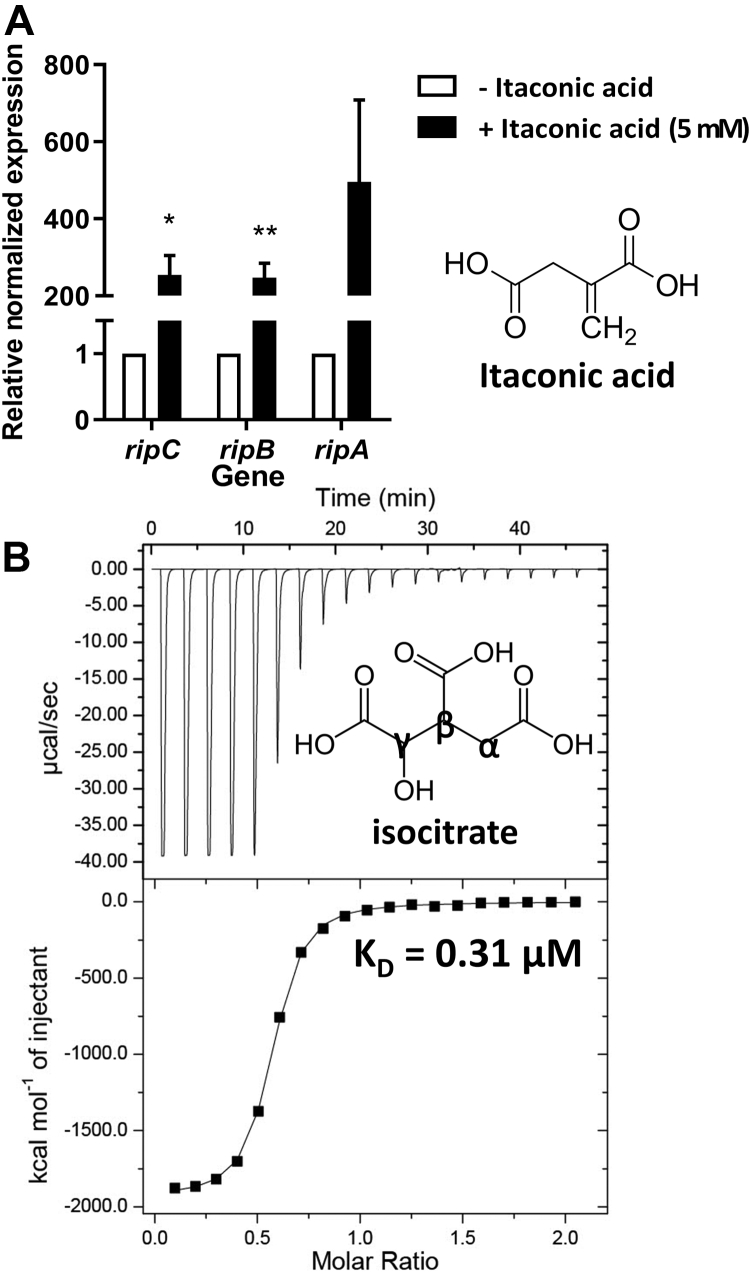
**Screening of the cognate ligand for RipR.***A*, the transcriptional levels of *ripC*, *ripB*, and *ripA* in the *S*. Typhimurium SL1344 WT were analyzed with quantitative real-time PCR (qRT–PCR). The strain was cultured in LB medium with or without 5 mM itaconic acid for 4 h. The expression value was normalized using samples cultured in LB broth without itaconic acid. Transcription of target genes was normalized to *gyrB*. Error bars represent SD values calculated from three replicate experiments, and the *p* value was calculated with Student’s *t* test. ∗*p* < 0.05; ∗∗*p* < 0.01. *B*, ITC graph for the titration of isocitrate to RipR RD. The ligand injection profile (raw data; *top*) and the calculated heat/enthalpy change for each ligand injection (*bottom*) are shown in the graph. The stoichiometry value (N) and KD of RipR RD with isocitrate were calculated as 0.5 sites and 0.31 μM, respectively. ITC, isothermal titration calorimetry; RD, regulatory domain.

To determine whether RipR of *S.* Typhimurium is responsive to the itaconic acid treatment, we treated *S.* Typhimurium with itaconic acid and investigated the transcription of the *ripCBA*, whose transcription is induced by RipR. The quantitative real-time PCR (qRT-PCR) results showed that the treatment of itaconic acid to the bacteria increased the expression levels of *ripCBA* by over 200-fold, confirming the role of RipR (18, 19) (Fig. 3*A*). Since RipR plays a significant role in itaconic acid stress in *S*. Typhimurium, itaconic acid and its structural analogs were suggested to be the cognate ligands for RipR RD.

To seek the physiological ligand for RipR, we selected itaconic acid, together with its structural analogs, isocitrate, succinic acid, malic acid, oxaloacetate, and *cis*-aconitate, which are the dicarboxylic or tricarboxylic acids found in the TCA cycle. We measured the binding affinities of the compounds to the RipR RD proteins using isothermal titration calorimetry (ITC) in 20 mM Hepes (pH 7.0). The ITC thermograms showed that isocitrate is strongly bound to the RipR RD with a submicromolar K_d_ value (0.31 μM) and a stoichiometry of 0.5 binding sites per RD monomer (Fig. 3*B*). The heat from the binding of isocitrate was large (Fig. 3*B*), which was unusually high and further indicated that many interactions were newly generated by isocitrate binding. In contrast, itaconic acid, succinic acid, malic acid, oxaloacetate, and *cis*-aconitate did not give compelling results for binding to the RipR RD in the ITC experiments (Fig. S3).

**Figure 4:**
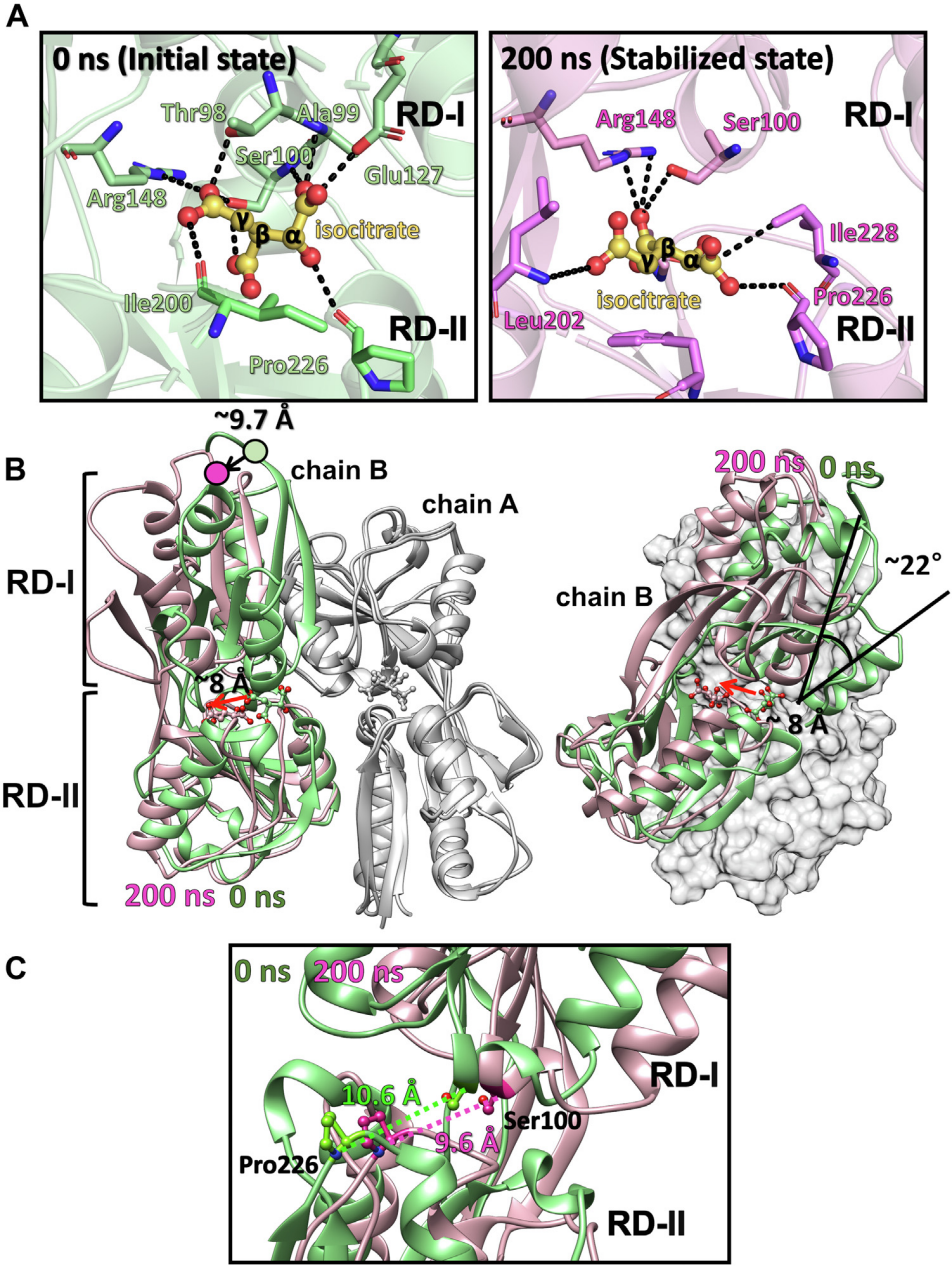
**Conformational changes upon isocitrate binding.***A*, a close-up view of the ligand-binding site. The residues surrounding the isocitrate are shown as a *stick model*, and the structures of RipR RD are shown as a *cartoon model*. The RD-I and RD-II of RipR RD are indicated. Critical residues are shown separately with observations from the same direction and same scale. *Left*, isocitrate-docking structure of the RipR RD, *pale green* (RD-I) and *green* (RD-II). *Right*, 200 ns MD simulation structure of the RipR RD, *light pink* (RD-I) and *light purple* (RD-II). The residues are shown as *sticks*. The isocitrate in the RipR RD is shown as a *yellow ball* and *stick*. The interactions between residues and isocitrate molecules are represented with *dotted lines*. *B*, structural superposition between chain B of 0 ns isocitrate-bound RipR RD (*pale green*) and chain B of 200 ns isocitrate-bound RipR RD (*light pink*). The length of the movements of the DBD connection points and the isocitrate are shown as *arrows* with each value (*circle*; DBD connection points, *black arrow*; DBD connection movement, *red arrow*; isocitrate movement). Each chain A in both dimers is colored *gray*, and chain B of the 0 ns isocitrate-bound dimer and chain B of the 200 ns isocitrate-bound dimer are colored *pale green* and *light pink*, respectively. *Left*, the angle (∼22°) between chain B in the 0 ns isocitrate-bound RipR RD (*pale green*) and chain B in the 200 ns isocitrate-bound RipR RD (*pink*) is shown as *black lines* with a value. Each chain A in both dimers is colored *gray* with surface representations. *C*, a close-up view of RD-I and RD-II at the ligand-binding site in residues Ser100 and Pro226. The distances of the 0 ns and 200 ns structures are shown as *dotted lines* colored in *green* and *pink*, respectively. The residues are drawn in *ball* and *stick* format with values shown. DBD, DNA-binding domain; RD, regulatory domain.

**Figure 5:**
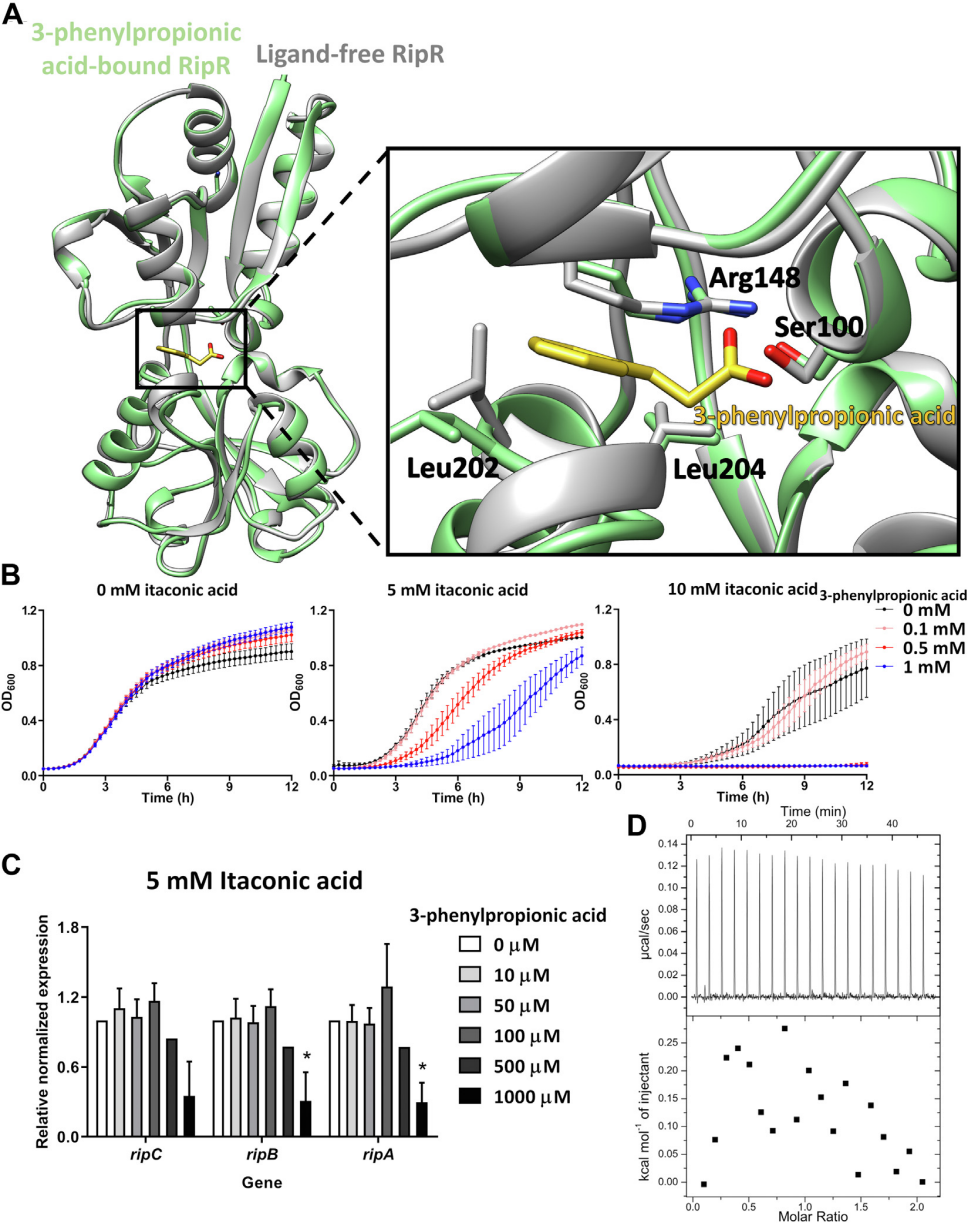
**Binding of 3-phenylpropionic acid to RipR RD.***A*, structural superposition between 3-phenylpropionic acid–bound RipR RD (*pale green*) and ligand-free RipR RD (*gray*). (rmsd = 0.603 Å) The 3-phenylpropionic acid is shown as a *stick* (*yellow*) and the interacting residues are represented as *sticks*. The *black rectangle* indicates the interacting residues for 3-phenylpropionic acid and RipR RD in a close-up view. *B*, growth curves of WT *S*. Typhimurium SL1344 in LB medium with various concentrations (0, 0.1, 0.5, and 1 mM) of 3-phenylpropionic acid were measured by A_600_. The mean and SEM values were calculated from three replicate experiments. *C*, the transcriptional levels of *ripC*, *ripB*, and *ripA* in the *S*. Typhimurium SL1344 were analyzed with quantitative real-time PCR (qRT-PCR). The strain was cultured in LB medium for 4 h with 5 mM itaconic acid at the indicated concentrations of 3-phenylpropionic acid. The expression values were normalized using samples cultured in LB medium containing 5 mM itaconic acid without 3-phenylpropionic acid. Transcription of target genes was normalized to *gyrB*. Error bars represent SD values calculated from three replicate experiments, and the *p* value was calculated with Student’s *t* test. ∗*p* < 0.05. *D*, the ITC thermograms for the titration of the isocitrate to RipR RD in the presence of 3-phenylpropionic acid in both ligand and macromolecule are displayed with the ligand injection profile (raw data; *top*) and the calculated heat/enthalpy for ligand injection (*bottom*). The 3-phenylpropionic is added first and then the sample is titrated with isocitrate. ITC, isothermal titration calorimetry; RD, regulatory domain.

### Conformational change of RipR upon isocitrate binding by MD simulation

To further investigate isocitrate binding in the RipR RD, we attempted to solve the complex structure with isocitrate. Unfortunately, isocitrate seemed to hamper the cocrystallization of the protein and did not allow binding by soaking into the ligand-free crystals. We instead docked an isocitrate molecule on the putative ligand-binding site of the RipR RD *in silico* by AutoDock Vina in PyRx (32) (Fig. S4*A*). The Gromacs program further minimized the energy of the docked structure, which depicted the isocitrate-bound ligand-binding site (Fig. 4*A*). The isocitrate molecule interacts with the side-chains of Glu127, Ile200, Thr98, Ala99, Ser100, Arg148, and Pro226 on the solvent-accessible ligand-binding pocket of RipR RD in the docked structure (Fig. S4*B*). In particular, Arg148 makes ionic interactions with a carboxylic acid moiety of isocitrate, as predicted by structural comparison to the *cis*-*cis*-muconate–bound BenM structure (Figs. 2*B* and 4*A*). When the *in-silico* docking model was compared with the ligand-free crystal structure, substantial structural changes were not observed, which was not surprising because the AutoDock Vina and the energy minimization function in Gromacs did not allow a large conformational change of the receptor proteins (Fig. S4*A*).

When we compared the elution volumes of the RipR RD in the buffer with and without isocitrate in the size-exclusion chromatography, isocitrate increased the elution volume of the RipR RD (Fig. S5). These findings indicate that isocitrate binding makes the conformation of RipR RD more compact, as observed in many ligand-bound LTTR RDs. To analyze the structural change of the RipR RD dimer upon isocitrate binding, we performed the molecular dynamics (MD) simulation with the docked structure using the Gromacs program, which is widely used in MD for biomolecules (33). The molecular motions of the protein and the ligand were largely stopped in 200 ns during the MD simulation process. According to the MD simulation result, the isocitrate molecule in a subunit (chain B) was moved into the interior of RD-II by 8 Å (Fig. 4*B*), while the isocitrate molecule in the other subunit (chain A) of the RD dimer remained in the solvent-accessible ligand-binding pocket. A large rotational motion was observed in the chain B subunit when the chain A subunit was superposed as the reference, which may explain the high released heat upon isocitrate binding in the ITC experiments. The rotational motion resulted in a 9.7 Å movement of the N-terminal residue of the RD (Val92) in the MD simulation, which is connected to the DBD in the full-length model of RipR (Fig. 4*B*). These findings suggest that isocitrate binding to the RD domains induces the transition from the inactive conformation to the active conformation by the movement of the DBDs in the tetrameric assembly of full-length RipR.

We analyzed the MD simulation results focusing on how the changes in the isocitrate binding mode resulted in the conformational change of the RD domain. The α- and γ-carboxylic groups and the hydroxyl group of isocitrate mediate the interaction between RD-I and RD-II. The α-carboxylic acid group of isocitrate (pKa ∼ 7.0) formed hydrogen bonds with the backbone or side-chains of RD-I Ala99 and Glu127 and the α-hydroxyl group of isocitrate with the carbonyl group of Pro226 in RD-II. The γ-carboxylic group formed hydrogen bonds with RD-I Ser100, Arg148, and RD-II Ile200 (Fig. 4*A*). However, the isocitrate-mediated RD-I and RD-II interactions were shifted in the 200 ns structure, resulting in a rotational movement of the protomers in the dimeric assembly. The angle between one protomer and the other protomer of the isocitrate-bound RipR RD dimer was decreased by ∼22° compared to the starting structure at 0 ns (Fig. 4*B*). The carboxylic group at the α position makes interactions with Pro226 and Ile228 only in RD-II, and the γ-carboxylic group interacts with Leu202 of RD-II with the ionic interaction between the β-carboxylic group of isocitrate and Ser100 and Arg148 in RD-I (Fig. 4*A*). The distance of RD-I and RD-II at the ligand-binding site (10.6 Å between RD-I Ser100 and RD-II Pro226 Cα atoms) at 0 ns was decreased at the final structure at 200 ns (9.6 Å between RD-I Ser100 and RD-II Pro226 Cα atoms), representing a typical ligand-dependent closing motion of the RDs (Fig. 4*C*).

### 3-Phenylpropionic acid as a RipR inhibitor

A BLAST search revealed that the *E. coli* HcaR gene has the highest sequence similarity (54.4%) to *S.* Typhimurium RipR. In *E. coli*, the LTTR HcaR recognizes hydroxycarboxylic acids, such as 3-phenylpropionic acid and its derivatives, regulating the genes of HcaA1, HcaA2, HcaC, HcaD, and HcaB (34, 35, 36) (Figs. 1*A* and S1). Although HcaR and RipR show different physiological responses, RipR and HcaR have the carboxylic acid group as the common moiety of their cognate ligands (36). Moreover, the Alphafold 2–predicted HcaR RD structure showed a high structural similarity to RipR RD, including the ligand-binding site–lining residues (Figs. S2 and S6). Thus, we tested whether 3-phenylpropionic acid is bound to RipR. The ITC experiment showed that the binding of 3-phenylpropionic acid to RipR RD (K_d_ value = 5.22 μM) released less heat than those from the isocitrate binding (Figs. S7 and 2*C*). These results suggested that 3-phenylpropionic acid binds to RipR RD with a less extensive structural change upon ligand binding than isocitrate.

To examine the binding mode of 3-phenylpropionic acid to the RipR RD, we determined the crystal structure of the RipR RD in complex with 3-phenylpropionic acid at 2.8 Å resolution. 3-phenylpropionic acid was bound in the ligand-binding site between the intersubdomain space (Fig. 5*A*). The carboxylic moiety of 3-phenylpropionic acid was near residues Ser100 and Arg148, and the phenyl ring was surrounded by Leu202 and Leu204 (Fig. 5*A*). Comparing the 3-phenylpropionic acid–bound structure of RipR and the benzoic acid–bound structure of BenM, the positions of the phenyl ring moiety and carboxylic moiety were similar. The phenyl ring moiety was captured by Leu202 and the carboxylic moiety interacted with Ser100 and Arg148 (Fig. S8). However, we found that the binding of 3-phenylpropionic acid did not substantially change the conformation of the RipR RD structure when it was superposed on the ligand-free structure (Fig. 5*A*). These findings suggest that 3-phenylpropionic acid interfered with the RipR function in itaconic acid resistance but did not activate RipR function.

To test whether the inhibitory function of 3-phenylpropionic acid against itaconic acid stress occurred, we cultured *S*. Typhimurium in the presence of itaconic acid and/or 3-phenylpropionic acid. Bacterial growth was not significantly inhibited at concentrations below 1 mM itaconic acid in the absence of 3-phenylpropionic acid (Fig. 5*B*). Interestingly, 3-phenylpropionic acid alone slightly increased the growth rate of the bacteria without itaconic acid treatment (Fig. 5*B*). Notably, the cotreatment of 10 mM itaconic acid and 0.5 or 1 mM 3-phenylpropionic acid completely shut down the bacterial growth (Fig. 5*B*). We further investigated the transcriptional levels of the *ripCBA* by 3-phenylpropionic acid treatment of the bacteria in the presence of 5 mM itaconate in the growth media. We found that the treatment of 1 mM 3-phenylpropionic acid to bacteria decreased the transcriptional levels of *ripCBA* by qRT-PCR (Fig. 5*C*). The results demonstrated that 3-phenylpropionic acid is an inhibitor of RipR that reduces the transcriptional activity of RipR in response to itaconic acid stress.

To further analyze how 3-phenylpropionic acid affects the binding of isocitrate to the RipR RD protein, we measured the binding affinity of isocitrate to the RipR RD protein in the presence of 3-phenylpropionic acid by using the ITC. We performed the ITC experiment by adding 3-phenylpropionic acid to both sample cell containing the RipR RD protein and injection syringe containing 300 μM isocitrate. No apparent binding enthalpy of isocitrate to the RipR RD protein was detected (Fig. 5*D*), in sharp contrast to the large binding enthalpy of isocitrate in the absence of 3-phenylpropionic acid (Fig. 3*B*). Since the large binding enthalpy of isocitrate also imply the structural change of RipR RD, our findings suggest that 3-phenylpropionic acid inhibits both the isocitrate binding and the ligand-induced activation of RipR.

## Discussion

**Figure 6:**
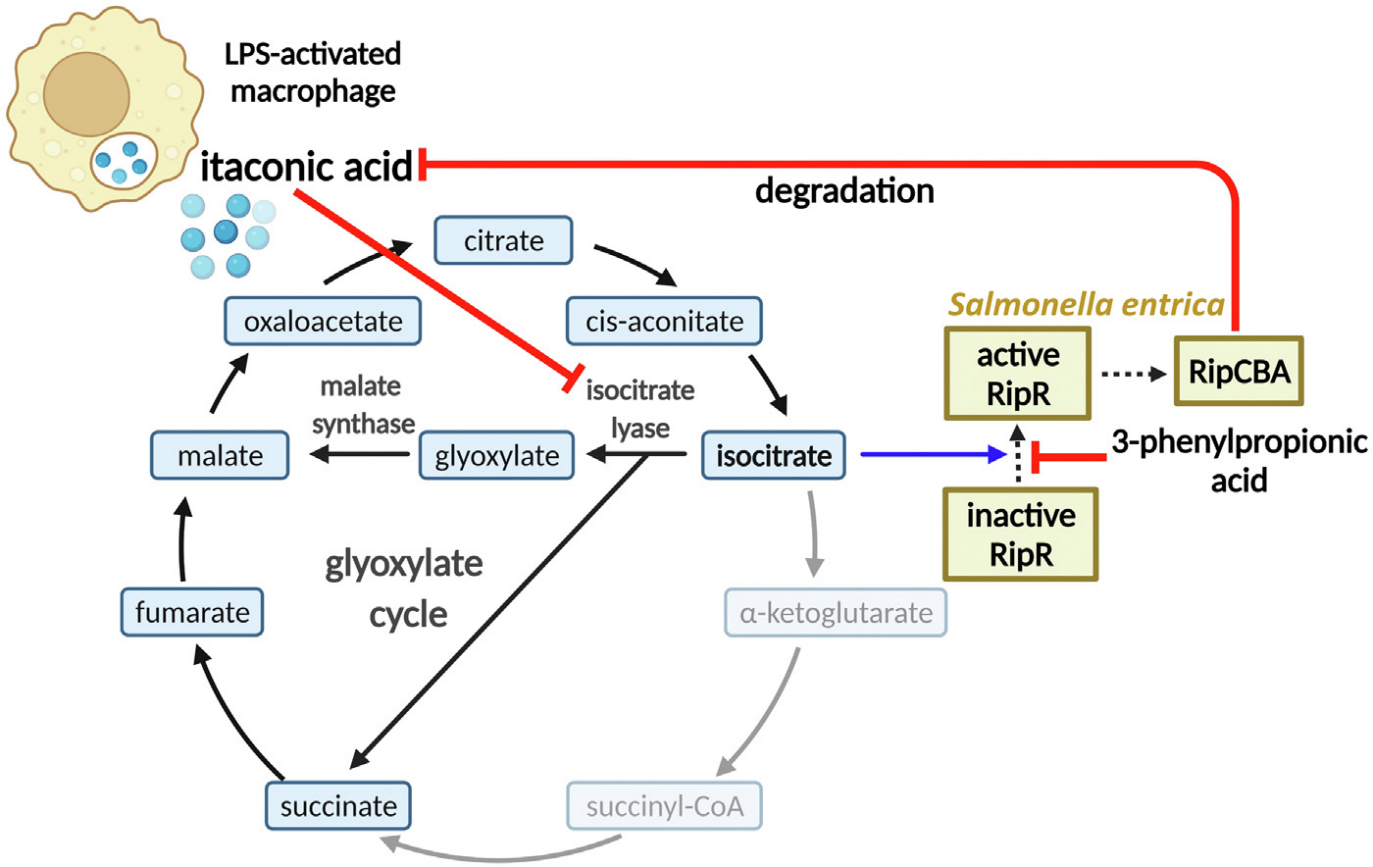
**Proposed mechanisms of RipR in defense against itaconic acid stress.** Schematic diagram of the roles of the LTTRs (*light yellow boxes*) RipR and RipCBA. LPS-activated macrophages produce itaconic acid in their phagosomes. *Salmonella* employs the glyoxylate cycle in the phagosome environment, and the function of isocitrate lyase in the glyoxylate cycle becomes critical for bacterial survival. Itaconic acid inhibits *Salmonella* isocitrate lyase, accumulating isocitrate in the bacterial cytosol in a competitive inhibitory manner. Bacterial RipR recognizes isocitrate and induces the expression of *ripCBA*, which decomposes the cellular itaconic acid to resume the glyoxylate cycle to survive. The natural compound 3-phenylpropionic acid inhibits RipR function, which makes the bacteria vulnerable to itaconic acid stress. The *black* and *gray arrows* indicate the glyoxylate cycle and tricarboxylic acid cycle, respectively. The *blue arrow* indicates that RipR recognizes isocitrate as a ligand to induce the expression of RipCBA. The suppression of the activity is indicated by *red lines*. The activation of the activity is indicated by *dotted lines*. LPS, lipopolysaccharide; LTTR, LysR-type transcriptional regulator.

Itaconic acid exhibits antimicrobial activity by inhibiting isocitrate lyase, which is the crucial and first enzyme of the glyoxylate cycle by converting isocitrate into glyoxylate and succinic acid (10). The bacterial RipR saves the glyoxylate cycle from itaconic acid stress by inducing the bacterial itaconic acid–degradation enzyme (18). In this study, we determined the crystal structure of *S. enterica* RipR RD in its dimeric form. We revealed that isocitrate was the strong ligand causing the structural change of the RipR RD into the active conformation by ITC and *in silico* simulation studies. We also found 3-phenylpropionic acid as an inhibitor of RipR at the molecular and the cellular levels (Fig. 6).

Isocitrate is the compound at the branching point between the TCA cycle and glyoxylate cycle. Under carbon source–limiting conditions, isocitrate dehydrogenase in the TCA cycle is inhibited and isocitrate lyase in the glyoxylate cycle is instead activated (13). Since it is expected that the inhibition of isocitrate lyase by itaconic acid increases the cellular isocitrate level, which is the substrate of isocitrate lyase in the glyoxylate cycle, the recognition of isocitrate by RipR is a reasonable response to cope with the abnormal metabolic situation caused by itaconic acid (Fig. 6). Why does RipR recognize isocitrate, but not itaconic acid? We noted that itaconic acid has many structural analogs in its metabolites and does not have a distinguishing moiety to make a possible polar interaction with its receptor proteins. In contrast, isocitrate has structural feature that can be specifically recognized by enzymes, such as isocitrate lyase and isocitrate dehydrogenase. Thus, we speculate that RipR has evolved to recognize isocitrate but not itaconic acid.

Since the glyoxylate cycle is central in the metabolism of pathogenic species including plants, bacteria, and fungi, isocitrate lyase is the current inhibition target for controlling diseases related to pathogens. Although the glyoxylate cycle is present in some animals, mammals including humans do not perform the glyoxylate cycle (37). In this study, we discovered 3-phenylpropionic acid as a potential inhibitor of RipR action in *Salmonella* in the presence of itaconic acid by interfering with the isocitrate binding to RipR RD (Fig. 6). 3-Phenylpropionic acid belongs to the class of organic compounds known as phenylpropanoids (35). Furthermore, it is among the most ubiquitous aromatic-compound catabolic systems (34, 35, 38). 3-Phenylpropionic acid has been identified as a volatile constituent of grapes and has a wide variety of uses including applications in cosmetics, food additives (wine making, aging, storage), and pharmaceuticals to control contaminating bacteria (39). Thus, our study may add the role of 3-phenylpropionic acid in controlling *Salmonella* species in food by inhibiting normal bacterial metabolism by activating itaconic acid actions.

We next noted that the RipR RD structure shares the residues lining the putative ligand-binding sites with many LTTR RDs, such as BenM and HcaR, whose functions are not directly related to the known function of RipR in the glyoxylate cycle. The sequence identity of RipR RD to RDs of BenM and HcaR (35.0% and 38.4%) is not high enough to consider them functional orthologs (Fig. S2). However, the lining residues that interact with the ligands are shared. These findings suggest that they have a common ancestral LTTR that could recognize carboxylic acids (Fig. S8).

In this study, we investigated the structure and ligand of RipR from *S*. Typhimurium and the bacterial strategy for maintaining the homeostasis of their metabolic pathways from mammalian antimicrobial metabolites in terms of structural and evolutionary aspects. Furthermore, our findings suggest that the natural compound 3-phenylpropionic acid may be a good inhibitor for restraining pathogenic bacteria.

## Experimental procedures

### Protein expression and purification

The PCR-amplified RipR RD gene from *S. enterica* was inserted into the pProEx-HTa vector (Thermo Fisher Scientific) with a hexahistidine tag and the tobacco etch virus protease cleavage site at the N-terminus of the RipR RD. The plasmids were transformed into the *E. coli* BL21 (DE3) strain and subsequently cultured in LB medium containing 100 μg/ml ampicillin at 37 °C. The RipR RD protein was induced at an optical density at 600 nm (A_600_) of 1.0 by 0.5 mM IPTG with a subsequent 6-h incubation at 30 °C. The harvested cells were resuspended in 50 ml of lysis buffer composed of 20 mM Hepes (pH 7.0), 300 mM sodium chloride, and 2 mM β-mercaptoethanol. The cells were disrupted using a French press (Constant Systems Limited) at a pressure of 23 kpsi and were cleared by centrifugation at 19,000*g* for 30 min at 4 °C. The supernatant was loaded onto nickel-nitrilotriacetic acid affinity agarose resin (GE Healthcare) in lysis buffer. The RipR RD protein was eluted with 250 mM imidazole in lysis buffer. The hexahistidine tag was cleaved from the protein using tobacco etch virus protease and then further purified by flowing through an anion exchange chromatographic column (HiTrap Q column; GE Healthcare). The flow-through proteins were concentrated using a Vivaspin centrifugal concentrator (30 kDa molecular-weight cutoff) and loaded on a Superdex 200 HiLoad 26/600 column (GE Healthcare) in lysis buffer for size-exclusion chromatography. The final protein concentration was 15 mg/ml.

### Crystallization and data collection

The initial crystallization trials of the purified 3-phenylpropionic acid–bound RipR RD and ligand-free RipR RD protein were performed in a MOSQUITO automated crystal screening device at 14 °C with a sitting-drop vapor-diffusion method. The RipR RD crystals were obtained with the hanging-drop diffusion method under a reservoir solution containing 0.2 M sodium formate (pH 5.75) and 19% (w/v) PEG 3350 at 14 °C in a 15-well plate. The crystals of RipR RD in complex with 3-phenylpropionic acid were obtained under a reservoir solution containing 0.1 M sodium cacodylate trihydrate (pH 6.5) and 1.4 M ammonium sulfate at 14 °C in a 15-well plate. For data collection, the crystals of ligand-free RipR RD and 3-phenylpropionic acid–bound RipR RD were transferred to 2 μl of Cryo Mix 4 (CryoProtX^tm^) and 20% (vol/vol) glycerol, respectively, and incubated for 10 s. Then, crystals were flash-cooled in liquid nitrogen at −173 °C for data collection. The datasets were collected at wavelength of 1.04477 Å and 0.97957 Å on an ADSC quantum Q270 CCD detector in beamline 11C of the Pohang Accelerator Laboratory, Republic of Korea. The program HKL2000 was used to process, merge, and scale the diffraction datasets (40). Table S1 describes the data-collection statistics.

### Structural determination and refinement

X-ray diffraction data were processed using HKL2000 software (https://hkl-xray.com) (40). The structure of ligand-free RipR RD was determined by the molecular replacement method using the program MOLREP (41) in the CCP4 package (42). The model structure was generated by the programs AlphaFold 2 (24, 25) and ColabFold (43). The ligand-free RipR RD was refined using the software programs PHENIX refine (https://phenix-online.org) (44) and Coot (https://www2.mrc-lmb.cam.ac.uk) (45). The structures of 3-phenylpropionic acid–bound RipR RD were determined using the ligand-free structure. The final structures of RipR RD were refined using the PHENIX software suite (44).

### Size-exclusion chromatography with multiangle light scattering

The sizes of the RipR RD protein were assayed using size-exclusion chromatography with multiangle light scattering. A high-performance liquid chromatography pump (Agilent) was connected to a Superdex-200 10/300 GL gel filtration column (GE Healthcare) and a MALS instrument (Wyatt Dawn Heleos). The size-exclusion chromatography column was preequilibrated with buffer containing 20 mM Hepes (pH 7.0), 300 mM NaCl, and 2 mM β-mercaptoethanol for RipR RD. Bovine serum albumin (2 mg/ml) was used as the standard. RipR RD (2 mg/ml) was injected onto the column and eluted at a flow rate of 0.2 ml/min. The datasets were evaluated using the Debye model for fitting static light-scattering data, and refractive index peaks were presented in EASI graphs created using Astra V software (Wyatt Dawn Heleos).

### Isothermal titration calorimetry

MicroCal Auto ITC200 (Malvern Panalytical) at the Korea Basic Science Institute was used for the ITC experiments. All samples were prepared in a buffer containing 20 mM Hepes (pH 7.0), 300 mM sodium chloride, and 2 mM β-mercaptoethanol. The ligands, potassium threo-isocitrate, itaconic acid, sodium succinate, malic acid, sodium oxaloacetate, *cis*-aconitic acid, and 3-phenylpropionic acid were purchased from Sigma–Aldrich. RipR RD (30 μM) was prepared in the sample cell, and each ligand (300 μM) was loaded into a titrating syringe. The titrations were measured with 19 2-μl injections with 150-s spacing at 25 °C.

### Molecular docking and MD simulations

The molecular docking of the compounds used in the ITC analysis on the RipR RD ligand-binding pocket was conducted by AutoDock Vina in PyRx virtual screening software (https://pyrx.sourceforge.io) (32). To further refine the docked structures, we employed energy minimization and equilibrium procedures in the MD simulation program. The Gromacs software package (33, 46) was used for the MD simulation to evaluate RipR RD toward the binding structure of isocitrate and docking studies of the isocitrate molecules with the protein. The topology file for protein and Gromacs files were prepared using the CHARMM36m force field in CHARMM-GUI (47, 48). The structure was solvated in TIP3P water and neutralized by adding 150 mM KCl molecules. Protein and ligand molecules were merged for each system, solvated with TIP3P water molecules, energy minimized, and equilibrated. The system was subjected to energy minimization using the steepest descent algorithm. The minimized state was equilibrated with a 125 ps NVT simulation to attain the temperature of 310 K and a 125 ps NPT simulation for the target pressure of 1 bar. The resulting complex structure was regarded as the refined structure at 0 ns. The time step was set to 0.002 ps following a published protocol. The MD simulation was carried out for 200 ns, which was started at the endpoint of equilibration. The RMSD of the simulated structure was calculated from the trajectory data using the Gromacs tool.

### *Salmonella* strain and growth inhibition assay

*S. enterica* Typhimurium SL1344 was used in this study and grown at 37 °C on LB medium (Difco). The *S*. Typhimurium strain was cultured in LB medium overnight. Then, the cells were inoculated (1:100 ratio) into fresh LB media with various concentrations (0, 5, and 10 mM) of itaconic acid in a 24-well plate. After treatment with the given concentrations (0, 0.1, 0.5, and 1 mM) of 3-phenylpropionic acid, the plate was incubated at 37 °C. The A_600_ were measured with SpectaMax i3 Platform (Molecular Devices).

### RNA extraction and qRT-PCR

The *S.* Typhimurium strain was cultured in LB medium containing 0 or 5 mM itaconic acid at the given concentrations (0, 10, 50, 100, 500, and 1000 μM) of 3-phenylpropionic acid. After incubation of the bacterial cells for 4 h at 37 °C, total RNA was extracted using an RNeasy Mini Kit (QIAGEN) and its cDNA was synthesized using EcoDryTM Premix and random hexamers (Takara). The cDNA was mixed with 2× iQ SYBR Green Supermix (Bio-Rad) and 0.3 μM of each primer in a 20 μl reaction volume. The qRT-PCRs were performed in the CFX Connect Real-Time PCR detection system (Bio-Rad) using the primer sets listed in Table S2. The transcription level of *gyrB* was used for normalization.

## Data availability

The crystal structure of RipR and 3-phenylpropionic acid–bound RipR (residues 87–292) is deposited at the Protein Data Bank (PDB) with PDB ID 7V5V and 7XRO, respectively. PDB structure data tables are provided in Table S1.

## Supporting information

This article contains supporting information (49).

## Conflict of interest

The authors have no potential conflicts of interest to disclose.
