# Supplementary material for: Isocitrate binds to the itaconic acid–responsive LysR-type transcriptional regulator RipR in Salmonella pathogenesis
Source: J Biol Chem. 2022 Oct 2;298(11):102562. doi: 10.1016/j.jbc.2022.102562 (PMC9637912; doi:10.1016/j.jbc.2022.102562)
Supplement: Supplemental Figures S1–S8 and Tables S1, S2 [file mmc1.pdf]

The activation mechanism of the itaconic acid-responsive LysR-type transcriptional regulator RipR in the *Salmonella* pathogenesis

Nayeon Ki[1,2†], Jinsil Kim[1,2†], Inseong Jo[1,2], Yongseong Hyun[1,2], Sangryeol Ryu[1, 2*], and Nam-Chul Ha[1,2*]

[1] Department of Food and Animal Biotechnology, Department of Agricultural Biotechnology, and Research Institute for Agriculture and Life Sciences, Seoul National University, Seoul 08826, Republic of Korea

[2] Center for Food and Bioconvergence, Seoul National University, Seoul 08826, Republic of Korea

[†] These authors contributed equally to this work.

* To whom correspondence should be addressed: Nam-Chul Ha (e-mail: hanc210@snu.ac.kr) and Sangryeol Ryu (e-mail: sangryu@snu.ac.kr)

Present address for J.K.: Department of Food Science & Biotechnology, and Carbohydrate Bioproduct Research Center, Sejong University, Seoul 05006, Republic of Korea

Running title

Itaconic acid-responsive LysR-type transcriptional regulator RipR

Keywords

itaconic acid, LysR-type transcriptional regulator, isocitrate, glyoxylate cycle, 3-phenylpropionic acid

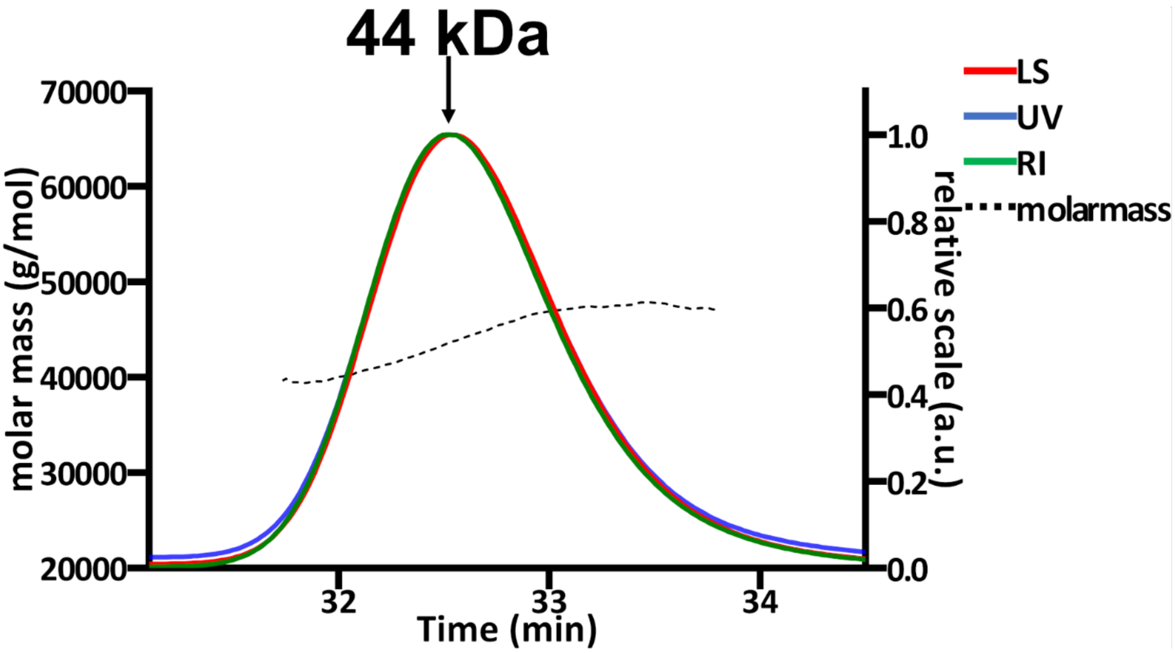

Supplementary Figure 1. The multiangle light scattering (MALS) of RipR

Molecular size of the purified RipR RD protein. Primary y-axis, molar mass determined using multiangle light scattering (MALS; black dotted line); secondary y-axis, protein concentration, assessed by measuring the light scattering (LS; red), the absorbance at 280 nm (UV; blue), and the refractive index (RI; green). x-axis, elution time from size exclusion chromatography. The estimated molecular mass of the purified RipR RD is indicated above the peak.

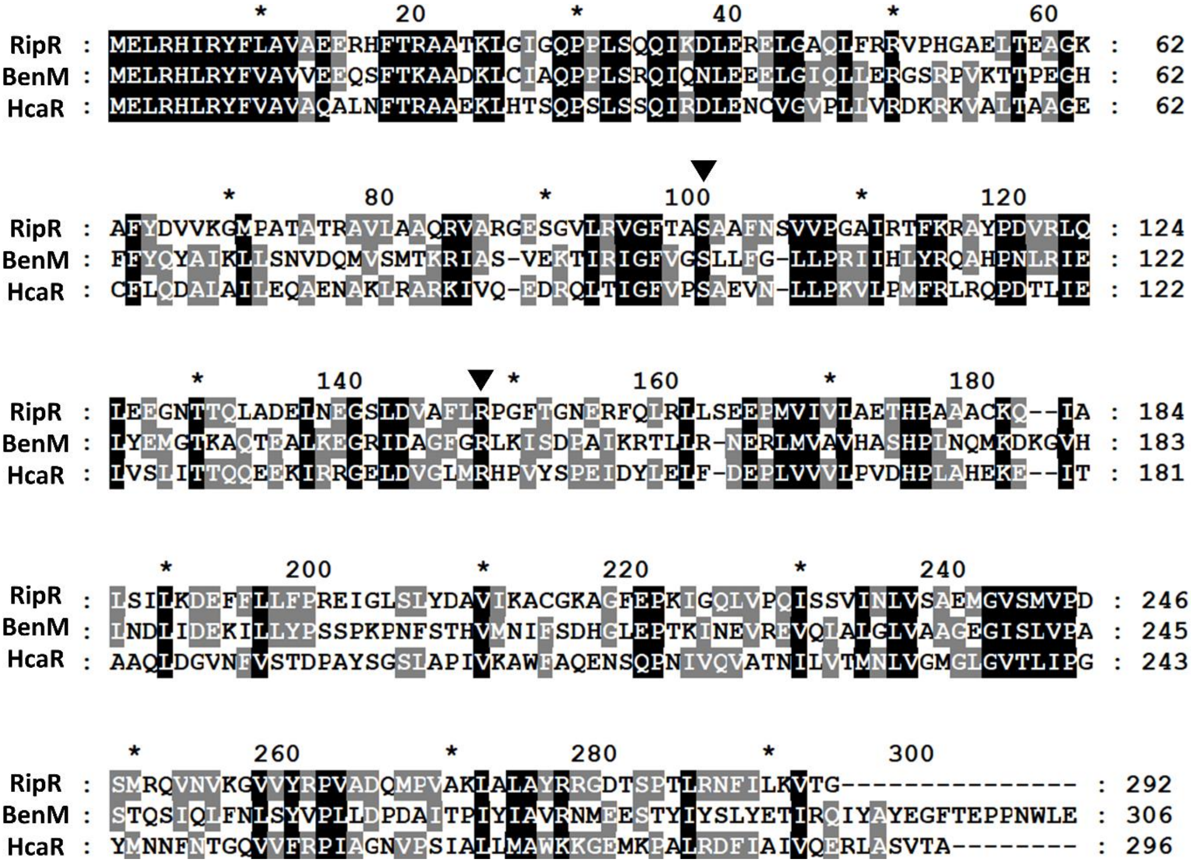

Supplementary Figure 2. Sequence alignment of RipR and its homologs

Sequences of RipR are aligned to its homologs: BenM, *Acinetobacter balylyi*; HcaR, *Escherichia coli*, by ClustalX (1). The conserved residues for recognition of the ligands are indicated by triangles.

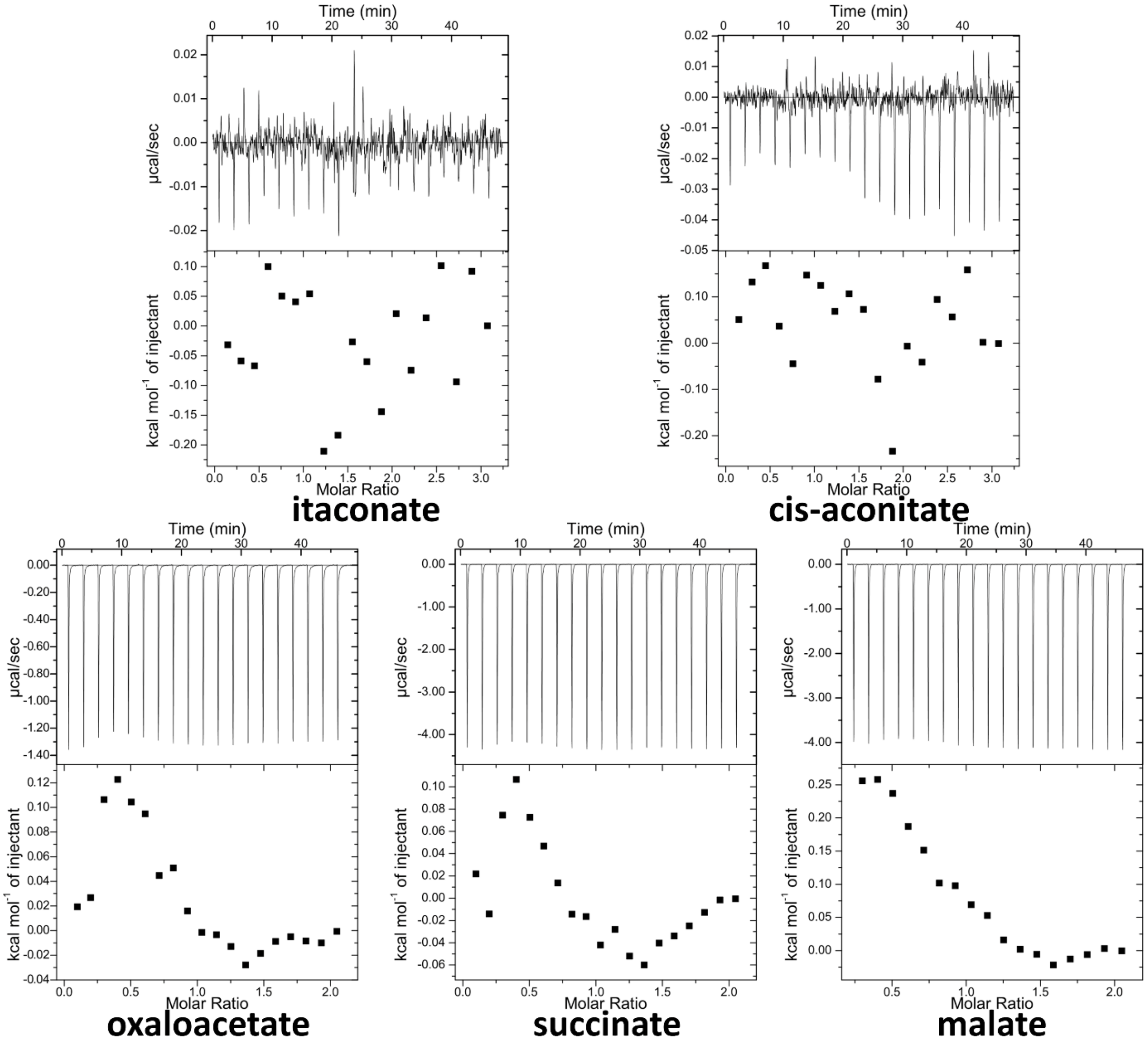

Supplementary Figure 3. ITC thermograms to screen the cognate ligand for RipR RD

The ITC thermograms for the titration of the listed structural analogs in the glyoxylate cycle to RipR RD are displayed with the ligand injection profile (raw data; Top) and the calculated heat/enthalpy change for each ligand injection (Bottom). Each ligand is mentioned below the ITC graph.

A

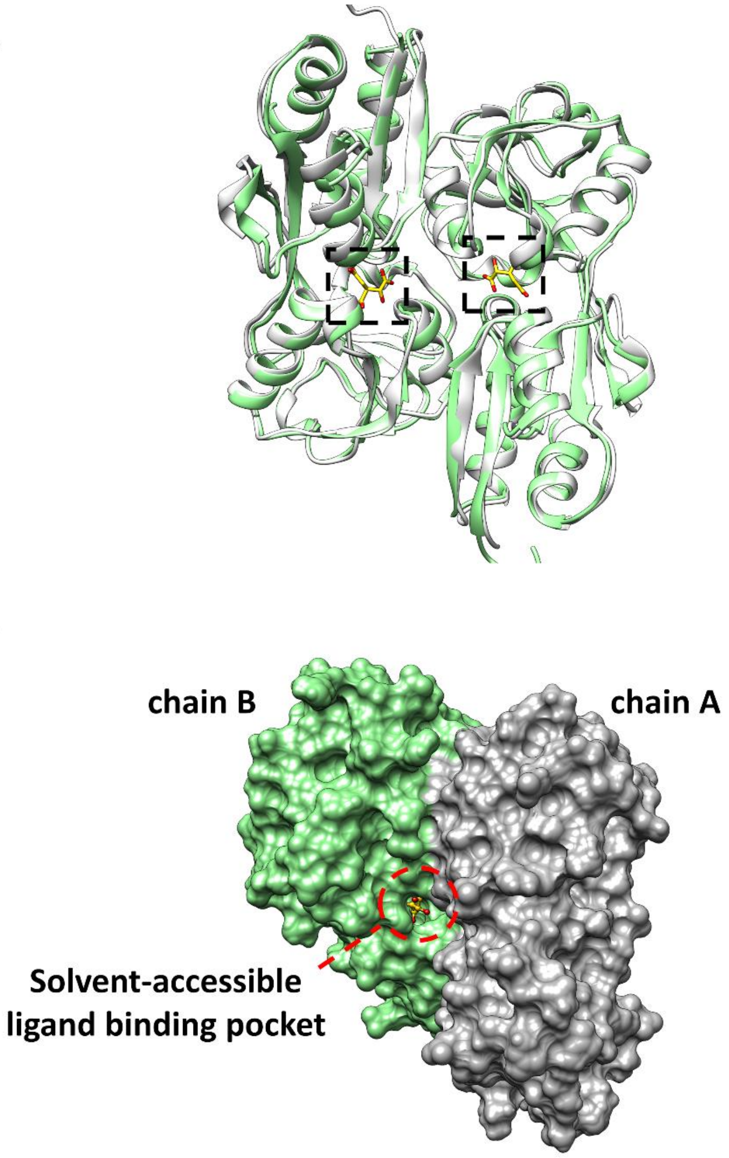

Supplementary Figure 4. The docked structure of isocitrate on the ligand-free RipR RD structure

A. Isocitrate molecules (yellow sticks) were first docked on the ligand-free RipR RD structure (gray ribbon) we determined in this study with PyRx. Then, the docked structure was further refined by the energy minimization and equilibrium procedures in the MD simulation program Gromacs (palegreen ribbon). The rmsd value between the PyRx-docked structure and Grmoacs-refined structure is 0.831 Å.

B. The surface representations of the Gromacs-refined complex structure. Each protomer is colored differently, and the bound isocitrate is shown as the yellow sticks. Note that the bound isocitrate is accessible from the external solvent.

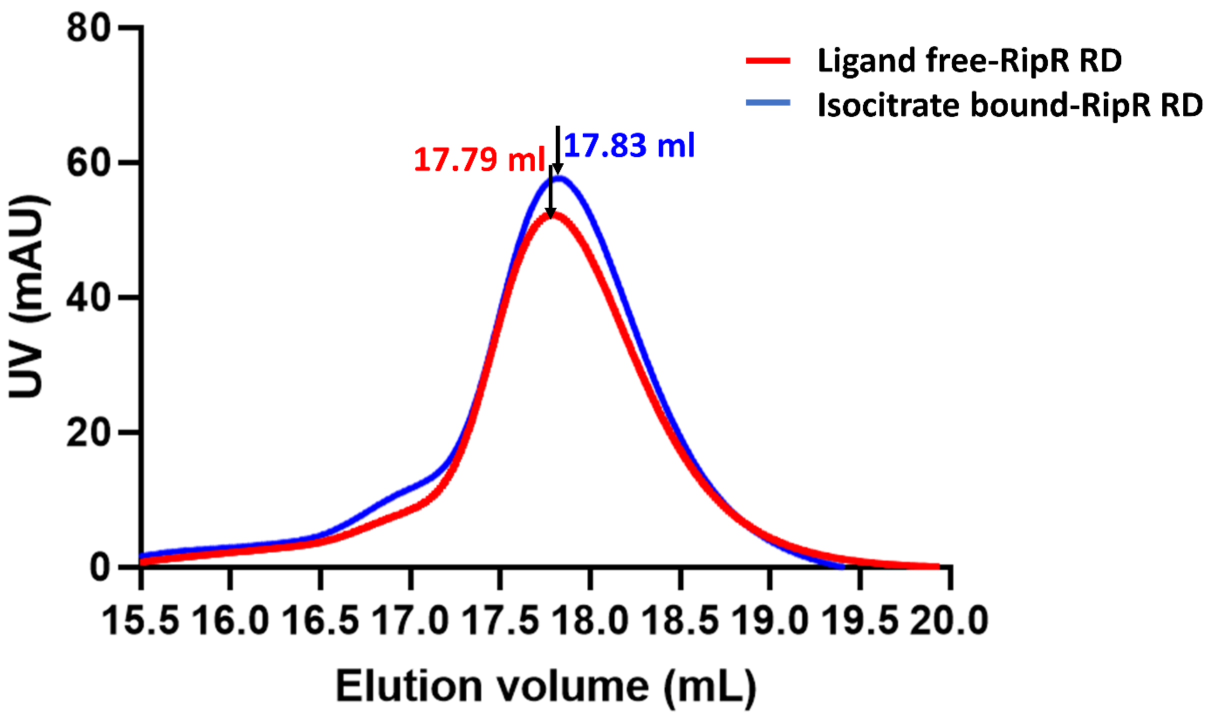

Supplementary Figure 5. Comparisons of Size Exclusion Chromatography (SEC) elution profiles.

The ligand-free RipR and isocitrate-bound RipR (165 μM) were injected on a Superdex 200 increase 10/300 GL column for profile comparison. Each elution volume of the main peak is indicated by a black arrow. The left y axis represents the absorbance at 280 nm and the x axis represents the elution volume. The scales for profiles in each graph are adjusted to be identical. The experiments were performed in triplicate.

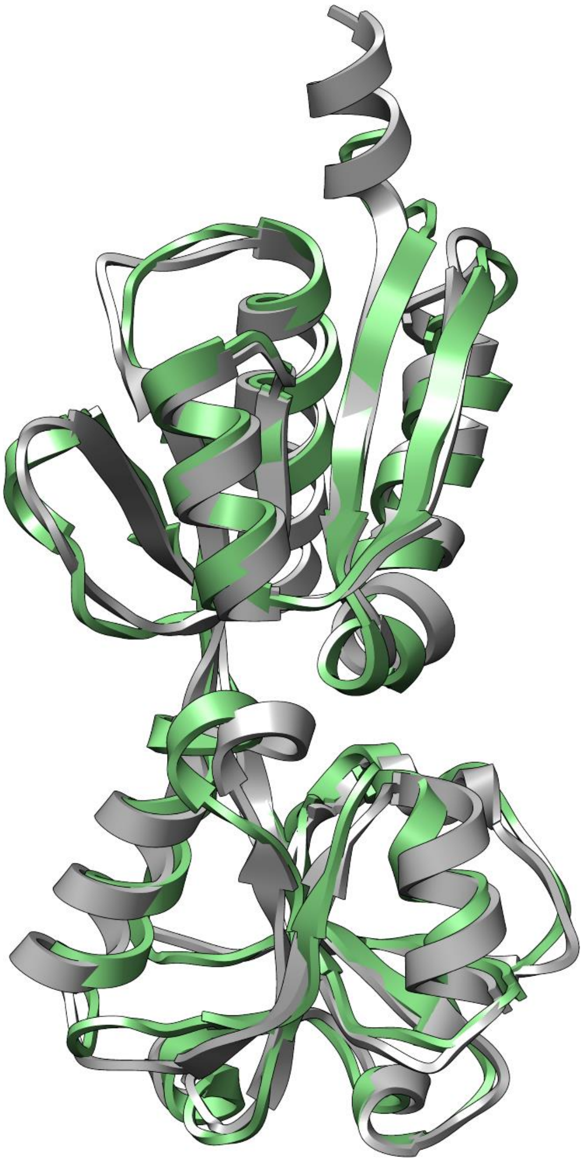

Supplementary Figure 6. Alphafold 2-predicted structure of *E. coli* HcaR RD.

The *E. coli* HcaR RD structure was predicted by Alphafold 2 using residues 80-296. For structural comparison, the predicted HcaR RD structure (gray) is superposed on the ligand-free RipR RD (palegreen) (rmsd = 1.444 Å).

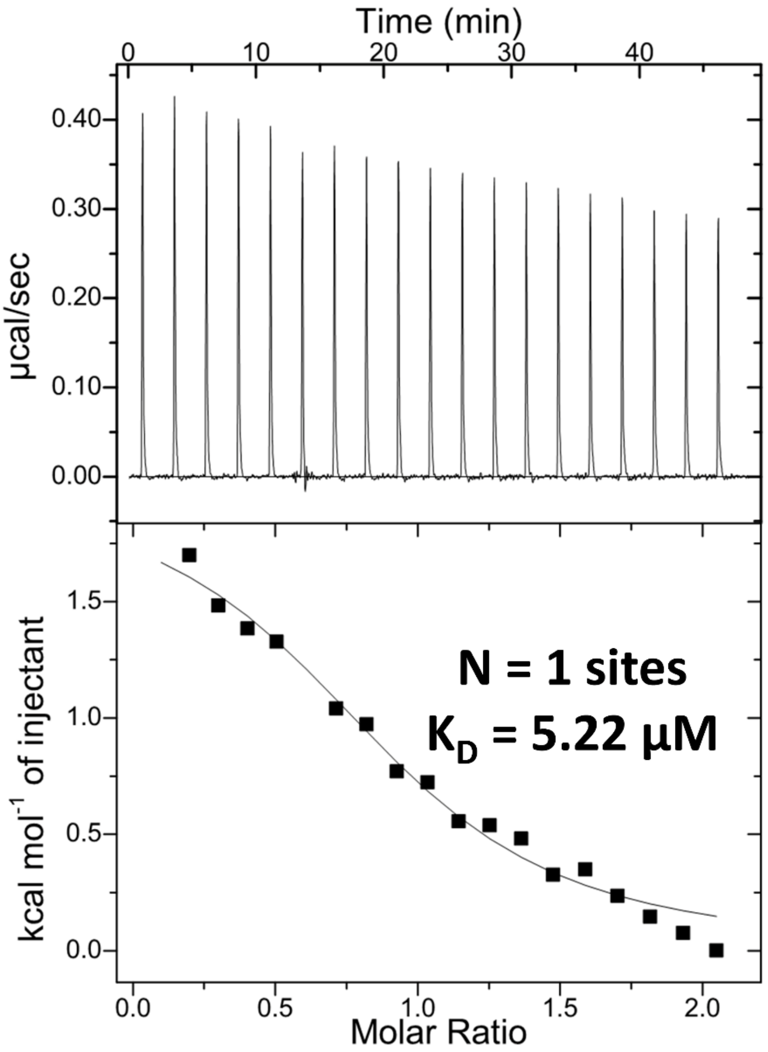

Supplementary Figure 7. The ITC thermogram of 3-phenylpropionic acid on the RipR RD protein

The ligand titration profile (raw data; top) and the calculated heat/enthalpy change for each titrataion (bottom) are shown in the graph. The stoichiometry value (N) and K_D of RipR RD with 3-phenylpropionic acid were calculated as one site and 5.22 µM, respectively.

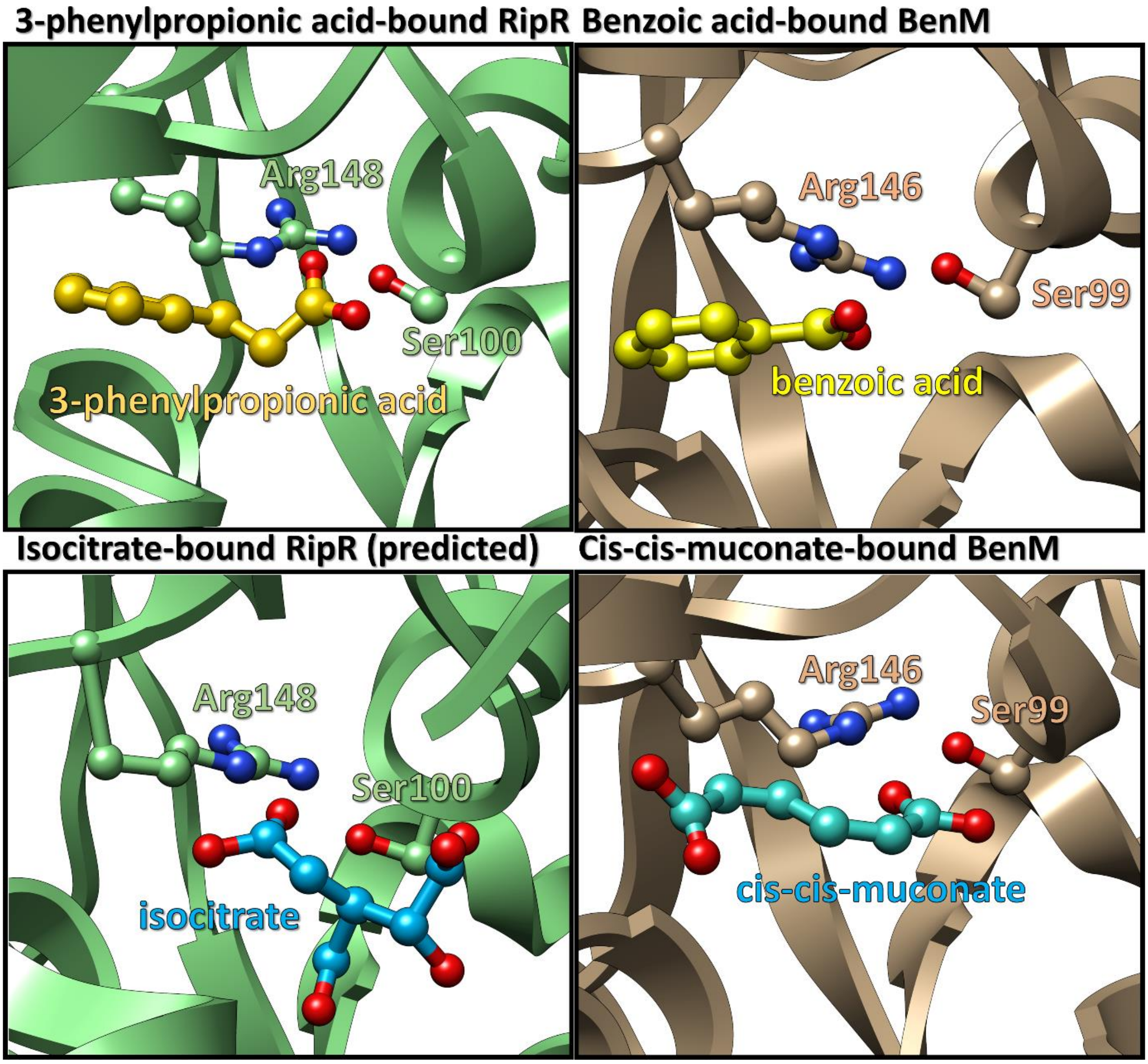

Supplementary Figure 8. The roles of Arg148 and Ser100 or their corresponding residues of RipR RD and BenM RD in recognition of the carboxylic group of the ligands.

The complex structures are shown as labeled above the figure. Arg148 and Ser100 of RipR and Arg146 and Ser99 of BenM are shown as stick representations. The bound ligands are shown as the stick representations: 3-phenylpropionyl acid (3-PP; gold), benzoic acid (yellow), isocitrate (blue), and cis-cis-muconate (cyan). Note that the isocitrate-bound RipR structure

was predicted by PyRx and Gromacs (see main text), while the others were determined experimentally.

Supplementary Table 1. **RipR X-ray diffraction and cell content analysis**

	Ligand-free RipR	3-phenylpropionic acid RipR
**Data collection**		
Beamline	PAL 11C	PAL 11C
Wavelength (Å)	1.00919	0.97957
Space group	$P2_1$	$P3_12$
Cell dimensions		
a, *b*, *c* (Å)	66.1, 72.4, 91.2	83.1, 83.1, 172.2
α, β, γ (°)	90.0, 104.6, 90.0	90.0, 90.0, 120.0
Resolution (Å)	50.00-2.20 (2.24-2.20)	50.00-2.80 (2.90-2.80)
Total No. reflections	42,095	17,472
R_{pim}	0.043 (0.159)	0.022 (0.448)
$CC_{1/2}$	0.990 (0.905)	1.000 (0.778)
I/σ(I)	17.6 (4.4)	31.6 (2.0)
Completeness (%)	98.7 (98.2)	99.25 (99.14)
Redundancy	6.1 (5.9)	20.1 (19.3)
**Refinement statistics**		
Resolution (Å)	39.26-2.37	28.70-2.80

No. of reflections	32,760	17,442
R_{work}/R_{free}	0.236/0.295	0.213/0.256
No. of total atoms	6,309	3,187
No. of protein atoms	6,279	3,148
No. of water atoms	30	26
No. of other atoms	0	13
Wilson B-factor	40.09	86.29
RMSD		
Bond lengths (Å)	0.002	0.003
Bond angles (°)	0.573	0.658
Ramachandran plot		
favored (%)	97.3	94.58
allowed (%)	2.46	5.17
outliers (%)	0.25	0.25
PDB ID	7V5V	7XRO

* The values in parentheses are for the highest resolution shell.

Supplementary Table 2. Primers used in this study

Primer	Sequence (5′-3′)	Reference
**qRT–PCR**		
gyrB-RT-F	ATATCGGCGACACGGATGAC	This study
gyrB-RT-R	CCTTCTTCCGGGTGAATCCC	
ripC-RT-F	ATCCCGACCAAACTCATCGC	
ripC-RT-R	GCGAGTATCAGAACCAGCCA	
ripB-RT-F	GCCAGCAAAACGGAATGGAA	
ripB-RT-R	ACAGGCGTTTGCTGAGTACA	
ripA-RT-F	CCTCCAGCTTTGCTGAATGC	
ripA-RT-R	CCGGAACTCTCAATCCCCTG	
